# Barriers and Facilitators to the Elimination of Asbestos Related Diseases—Stakeholders’ Perspectives

**DOI:** 10.3390/ijerph14101269

**Published:** 2017-10-22

**Authors:** Joanne Vincenten, Frank George, Marco Martuzzi, Peter Schröder-Bäck, Elizabet Paunovic

**Affiliations:** 1 Faculty of Health, Medicine and Life Sciences, School CAPHRI-Care and Public Health Research Institute, Department of International Health, Maastricht University, P.O. Box 616, 6200 MD Maastricht, The Netherlands; peter.schroder@maastrichtuniversity.nl; 2 World Health Organization Regional Office for Europe, the European Centre for Environment and Health, Bonn D-53113, Germany; georgef@who.int (F.G.); martuzzim@who.int (M.M.); paunovice@who.int (E.P.); 3 Faculty of Human & Health Sciences, Bremen University, Bremen D-28359, Germany

**Keywords:** asbestos, barriers, facilitators, evidence implementation

## Abstract

Despite sound scientific knowledge and evidence that any exposure to asbestos fibers in all of its forms, are carcinogenic to humans, its presence, use and trade is still substantial, including in the World Health Organization (WHO) European Region. Banning the production and use of all forms of asbestos, as recommended by the International Labour Organization (ILO) and WHO, has been proven as the most efficient evidence-based strategy to eliminate Asbestos Related Diseases (ARDs). To effectively move elimination of ARDs forward, attaining a greater understanding of key stakeholders perspectives was identified as an important action. The WHO Regional Office for Europe, the European Centre for Environment and Health, undertook semi-structured interviews, and follow-up discussions with diverse representatives dealing professionally with asbestos. The interview questionnaire was developed based on the current ARDs WHO Report, the Evidence Implementation Model for Public Health and categories of the theory of diffusion. Data were attained on three main questions within the interview questionnaire: (1) Identifying barriers to implementation of WHO evidence-based asbestos recommendations; (2) Describing roles of key stakeholders; and, (3) Proposing possible solutions. The results demonstrated use of sound and convincing scientific evidence along with economic evidence and facilitators can be used to achieve evidence-based policy development, and comprehensive diverse actions.

## 1. Introduction

Despite sound scientific knowledge and evidence that any exposure to asbestos fibers in all of its forms, are carcinogenic to humans, its presence, use, and trade is still substantial, globally and regionally, including in the World Health Organization (WHO) European Region [1,2,3]. Globally, more than 2 million metric tons of asbestos is still produced annually, with 99 percent generated by four countries—Russian Federation, China, Brazil, and Kazakhstan. Currently, 25 countries consume at least 1000 metric tons of asbestos per year, of which seven are in the WHO European Region (Belarus, Kazakhstan, Kyrgyzstan, Russian Federation, Turkmenistan, Ukraine, and Uzbekistan) [4,5].

Evidence-based strategies for the elimination of asbestos related diseases (ARDs) exist. Banning the production and use of all forms of asbestos as recommended by the International Labour Organization (ILO) and WHO, has been proven as the most efficient evidence-based strategy to eliminate ARDs [6]. Still, the safe removal and disposal of already existing and used asbestos is a continuous threat to human health, even after the introduction of the ban of the use of all forms of asbestos. Although many countries have stopped the use of asbestos for years or even decades, there are many old homes, schools, and public buildings that still contain asbestos insulation, flooring, ceiling tiles, shingles, siding, and other items. Asbestos that is in good condition, does not usually present a hazard. However, worn or damaged asbestos poses a great risk to the health and safety of humans, as fibers may flake off and become airborne, inhaled, and then embedded into the chest of those near by during renovation or demolition projects if professional safety measures are not consistently used [6]. To date, 37 Member States out of 53 in the WHO European Region have adopted partial or total asbestos ban legislations, while countries not in support of any form of ban are still using and or producing asbestos [6].

In addition to detecting diseases, establishing appropriate surveillance systems, and protection, while undertaking asbestos removal, appropriate replacement, and safe and efficient waste management should be consistently occurring throughout the WHO European Region, to address inequities created by harmful exposure to asbestos [7,8].

Currently, some of the countries that did not introduce the ban of the use of asbestos are challenging this recommendation, often using economic arguments. They argue that the introduction of the ban of the use of all forms of asbestos will have negative economic impacts on national, private sector, and household income at national and regional levels [4]. Yet, available evidence does not support this argument. The WHO recent report “Asbestos-Economic assessment of bans and declining production and consumption” [4], stressed two key findings:

(1) There are no observable mid-or-long-term negative economic impacts from bans or a decline in asbestos production or consumption at the country-level, and no observable persistent negative effects at the regional level;
(2) There are substantial and increasing costs associated with the continuing production and use of asbestos, with the potential to far outweigh the short-term economic benefits.

To effectively move elimination of ARDs forward, attaining greater understanding of key stakeholders perspectives and needs was identified as an important action. Therefore, the WHO Regional Office for Europe, the European Centre for Environment and Health (WHO/ECEH, Bonn, Germany), undertook in 2016 a stakeholder analysis.

The aim of this analysis was to attain deeper insights of key stakeholders opinions and perspectives across different sectors and policy fields regarding the promotion of evidence-based national policies for the elimination of ARDs and the potential influence and ability of the stakeholders concerned to make the change towards non-asbestos working and living environments. This stakeholder analysis contributed, in particular, to one of the commitments of the Parma Declaration to develop national programmes to eliminate ARDs by 2015 and reconfirmed in the Ostrava Declaration 2017 [9,10].

## 2. Materials and Methods

The research method was qualitative and explorative, combining interviews and discussions with stakeholders. The questionnaire for the semi-structured interviews was developed based on information from a current ARDs WHO report [6] and the Evidence Implementation Model for Public Health Systems that offers insights into barriers and facilitators of uptake of effective strategies [11]. Furthermore, categories of the theory of diffusion were taken into account in formulating the interview questions [12]. The questionnaire was drafted by the first author and reviewed and edited by the other authors. In the questionnaire, three themes were focused on:

(1) dentifying barriers to the implementation of WHO evidence-based asbestos recommendations;
(2) describing necessary and effective roles and actions of key sectors and stakeholders;
(3) proposing possible solutions, facilitators and next steps towards non-asbestos working and living environments, including policies for the elimination of ARDs.

Interviewees were identified by WHO/ECEH and contained a selected sample of country representatives, experts, intergovernmental organisations, nongovernmental organisations, and European Commission (EC) representatives, dealing with asbestos issues in their respective professional area. All of the interviewees gave their informed consent for inclusion before they participated in the interviews and the study was conducted in accordance with the Declaration of Helsinki and WHO protocol. Interviews were conducted using telephone or Skype by the first author. Language of the interviews was English or Russian (with the assistance of a Russian interpreter). The first interview conducted also served as a pre-test and adjustments for clarity were made accordingly. Confidentiality of interviews was communicated to the interviewee and agreed upon. Interviews were transcribed verbatim into a corresponding questionnaire template. Clarifications to the participant’s comments were obtained by e-mail where required.

Content analysis of the interviews was undertaken to provide concise summaries of participants’ responses. Findings were grouped corresponding to the three interview themes noted above [11,13].

A preliminary report of this analysis was circulated and presented to participants of the WHO meeting: “Assessing the economic costs of the health impacts of environment and occupational factors: the economic dimension of asbestos”, 18–19 May 2016, Bonn, Germany. The preliminary results were validated by a plenary session, question, and answer discussion period, working group discussions and a final plenary reporting session. The results of the working group and plenary discussions were integrated into a preliminary and final text to prepare this article.

## 3. Results

A total of 21 stakeholders from 18 organisations participated in the stakeholder analysis interview process. A further 24 country representatives from 12 WHO European Countries and 20 temporary advisors from 16 institutions participated in the above cited WHO asbestos meeting. Data from stakeholders are presented on the three main questions within the interview questionnaire.

### 3.1. Barriers to the Implementation of WHO Evidence-Based Asbestos Recommendations

#### 3.1.1. Leadership Barriers

Lack of political will, commitment, clear vision, goals, and targets to effectively address the negative impact of asbestos is often not identified, agreed upon or achieved by policy makers.

#### 3.1.2. Management and Collaboration Barriers

Introducing a total ban of the use of all forms of asbestos is a complex undertaking that requires the collaboration of many sectors and disciplines to achieve effective action. Often collaborators may take some of the tasks or actions within their remit, but responsibility to lead and manage the whole chain of events is lacking.

#### 3.1.3. Funding and Financing Barriers

Often financial and political interests are placed above health concerns, including the assumed important positive economic impact of the production, and marketing of asbestos on national, private sector, and household income. Currently the economic health costs of ARDs are predominately the burden of society (e.g., health insurance, pensions) and not industry. Costs for safe asbestos identification, removal, replacement, and waste management are high. Illegal removal of asbestos is underway (at reduced costs) that does not meet safety requirements, leaving the general public exposed to even greater health risks.

#### 3.1.4. Capacity Building Barriers

There is a lack of awareness, understanding and knowledge regarding the issue of the negative health impacts of asbestos. This includes how to effectively address the harmful effects of under-reporting the negative impacts of asbestos and knowledge gaps of the real economic dimension of the use and production of asbestos in parts of Europe. This lack of information, knowledge, and expertise to undertake and finance asbestos removal, safe waste disposal, and replacement with safe alternatives affects many countries at this time.

#### 3.1.5. Data Barriers

Data and registries on exposures, risk groups, mortality, morbidity, trends, short and long-term costs, essential to demonstrate the true impact of asbestos, are not readily available in many countries. Data are also not standardised in Europe to allow for cross-country comparisons. There is a need for evaluation methodologies, such as cost benefit analysis to support policy-solutions. Understanding when and how much asbestos needs to be removed, the associated costs of removal and replacement, and then how that compares to the negative health impact costs are needed.

#### 3.1.6. Evidence-Based Strategies Barriers

Banning the use of all forms of asbestos as recommended by the ILO and WHO can be a challenging strategy to adopt, implement and enforce due to its inherent complexity. Few Member States with no asbestos banning legislation are not in agreement with this recommendation, claiming that in their countries there is no evidence of negative impacts on health from all forms of asbestos. Several Member States stated that more and better knowledge and awareness are required to meet European Union (EU) regulations/directives for occupational health and safety of asbestos removal and building renovations.

#### 3.1.7. Context Setting Barriers

Currently, there is a lack of political will amongst policy-makers from several asbestos producing and using countries in banning asbestos as the most efficient way to eliminate asbestos-related diseases, as the benefits are only realised decades later and not during their immediate political term. Varying economic and social settings in countries create additional barriers to reducing the negative impact of asbestos. These include communities with: mines, natural forms of asbestos, extensive existence of asbestos in road infrastructure, and the majority of public and private buildings containing asbestos. Furthermore, research related to health is sometimes financed by the asbestos industry itself, and in some cases are used as questionable “evidence” for policy solutions. As well, an influx of migrant workers with limited language skills in their newly employed location may also lead to limited awareness or knowledge of asbestos risks and a challenge to monitor. This includes lack of training and training material not available in migrant workers native language and often no migrant worker occupational health protection.

#### 3.1.8. Visibility and Communication Barriers

Key messages to the public and decision-makers are lacking regarding: risks, impacts, revealing false claims, illegal trade, international conventions, and declarations not being upheld, prevention measures, safe removal, total real costs, and valid information sources.

### 3.2. Roles of Key Stakeholders

#### 3.2.1. Whole-of-Government and Whole-of-Society Approach

Interviewees and workshop participants expressed their strong opinion that a whole-of-government and whole-of-society approach would be required to successfully address all of the negative impacts of asbestos. This means that all levels of government and all related sectors, using cross-ministerial committees. Governments need to consider the long-term health effects to balance the immediate financial or political demands and interests asbestos appears to provide.

#### 3.2.2. Industry, Employers, Trade Unions, Employees, and Victims

Even in the countries that introduced the total ban of use of all forms of asbestos, removal of the existing asbestos is still potentially a significant risk to health if the appropriate measures are not applied. Having that in mind, the responsibility of the industry, both in asbestos producing and asbestos using countries, as well as in the countries with introduced bans, can not disregard including compensation funds for ARD victims. Industry also needs to share knowledge on safe removal practices and safe alternatives to asbestos to help build a win/win situation.

#### 3.2.3. Experts/Professionals, Intergovernmental and Nongovernmental Organisations

Stakeholders with knowledge and information should ensure evidence and good practices are shared, widely communicated, and advocated to increase their adoption, implementation, and monitoring. Research also needs to be accessible and widely communicated, including the use of plain language summaries, tailored to each target audience to help move research into practice and thereby support effective knowledge translation.

#### 3.2.4. Media, General Public and Champions of the Asbestos Cause

Communicators also have a strong role to bring greater attention to the issue and apply pressure to attain action to protect and safeguard workers and the society as a whole. They need to be engaged and empowered as key stakeholders in the process. Therefore, all of society has an important role to play to address the negative impacts of asbestos.

### 3.3. Proposed Solutions, Facilitators, and Next Steps to Address Indentified Barriers

#### 3.3.1. Leadership, Management, Collaboration and Funding Proposed Solutions to Address the Negative Impacts of Asbestos

Government plans, programmes, and policies to support early identification, removal, transition, and waste management of asbestos to new, safer, and efficient alternatives, with the involvement of all key stakeholders should be investigated. This should include investigation of funding schemes, such as a percentage of programme funds for victims’ compensation from asbestos producing industries, as already demonstrated in some Member States.

Establishment of an agency/task force/observatory/knowledge centre at the European level to provide support to countries for the adoption and implementation of evidence-based strategies to enhance awareness raising; capacity building; standardisation; and, consistency and stability of various approaches for diagnosis, surveillance, trends, policy approaches, funding, and budgeting models was proposed by stakeholders. This would include implementing more effective legislation to protect employees and the health of the general public, as well as, the identification of funding sources and should be discussed in collaboration with WHO, EC, European Agency for Safety and Health at Work (EU-OSHA), ILO, Organization for Economic Co-operation and Development (OECD) (see Table 1), and other lead agencies.

Key stakeholders and especially intergovernmental organisations and nongovernmental organisations should jointly advocate for a total ban on the use of all forms of asbestos throughout the WHO European Region. Economic arguments learnt from the Bonn workshop should provide additional evidence and proposals to support this action.

#### 3.3.2. Evidence-Based Strategies and Proposed Solutions to Address the Negative Impacts of Asbestos

The total impact costs of asbestos at the local, regional, and national levels, including long-term health consequences, asbestos identification, removal, replacement, and waste management are needed, and the cost responsibilities of all the parties should be calculated. This could include, short-mid-long term budgeting strategies, impact assessments, cost benefit analysis, such as cost of asbestos substitutes, and case examples of building costs with asbestos when compared to safer alternatives, as well as landfill management costs.

Collaboration with industry and other key stakeholders for effective and efficient strategies on identification, safe removal, transition to safe alternatives and waste management of asbestos need further investigation.

Further research on the effective use of new technologies and availability of low cost, safe, and easy to use alternatives should be conducted and communicate results widely.

More case studies and good examples of how countries are addressing the issue of asbestos, specifically including removal guidelines/legal criteria and cost examples of safe removal, waste management, and replacement schemes, as compared to the negative health impact costs could be shared.

#### 3.3.3. Data and Context Setting Proposed Solutions to Address the Negative Impacts of Asbestos

Standardised and harmonised data registration systems for ARDs in all of the countries to ensure effective monitoring should be put in place. Countries with long-established, effective data collection systems, could share methodologies for establishing and sustaining such data collection systems. The sharing of misinformation that are contrary to evidence and positions widely accepted and published by the majority of the scientific and expert community need to be immediately addressed. False statements are to be rejected and widely communicated.

#### 3.3.4. Visibility, Awareness Raising and Capacity Building Proposed Solutions to Address the Negative Impacts of Asbestos

Visibility, awareness, and education of all target audiences, from the general public, workers, professionals, and governments on such topics as early detection and risk group identification for youth and migrant workers exposed to asbestos, require improvement. Targeted tools and resources should be determined on evidence-based strategies and include better use of science.

Further to these proposed solutions and next steps, participants also provided specific suggestions WHO should undertake to facilitate actions to address the negative health impacts of asbestos. These are described in Table 1.

## 4. Discussion

Even though addressing the elimination of ARDs is a challenging issue within a complex system, use of sound and convincing scientific evidence along with different types of economic evidence and or facilitators can be used to achieve evidence-based policy development, and comprehensive diverse actions [14]. Exposure to all forms of asbestos causes high negative health impacts [15]. The substantial and increasing costs associated with the continued production and use of asbestos far outweigh the short-term and often only local economic benefits [4]. There is a trend that countries are moving away faster than before from asbestos use and this pattern needs to be maximised by sharing the lessons learned, including the mid- and long-term potential economic benefits after banning asbestos such as evolvement of sustainable industry and reduced health sector costs [4,16]. The discussion and conclusions that follow are based on the responses of the stakeholders.

Enhanced, early engagement and cooperation of diverse sectors and stakeholders throughout the entire process of adoption, implementation, and monitoring of evidence-based policies and programmes to eliminate ARDs, based on a whole-of-government and whole-of-society approach, will support the knowledge translation process, as seen in other evidence implementation research [11]. Overall, empowering a lead agency so initiatives and actions can be coordinated is essential [17]. With the case of asbestos, a lead role from the Ministry of Health, in providing the evidence on health effects, with cooperation and collaboration of multiple diverse ministries, including but not limited to: labour, trade, industry, environment, education, customs, financial affairs/economy, would be beneficial. To attain true partner participation, co-benefits to addressing the negative health impacts of asbestos need to be demonstrated to other sectors to secure their political and financial commitment.

Greater efforts to transfer evidence-based strategies and knowledge into practice are essential to reduce ARDs. Particularly, attention in synthesising, translating, and disseminating evidence into concise, clear, easy to understand, and convincing information is needed, such as, economic arguments designed for each specific target audience, context, and cultural setting [18]. This should include the standardisation and harmonisation of easy to use tools and resources that will maximise current technology and should be made available and be provided as part of an awareness raising and mentoring process with key stakeholders, to build capacity and broaden the network needed to address ARDs. In turn this should increase visibility, mentoring, and capacity building of all of the key stakeholders so greater awareness and knowledge is attained about the risks, prevention, and management of asbestos, including strengthening capacity for diagnosis and surveillance.

Well-established national cancer registers and national registers of occupational diseases are the key resources for registering asbestos-related morbidity. Awareness, diagnostic procedures, and criteria, notification and registration of ARDs vary between countries and are in need of strengthening and harmonisation. There is still a need to strengthen research on the prevention, diagnosis, and treatment of ARDs. The main areas for action include capacity building of health care providers for the appropriate diagnostics of ARDs, active surveillance of ARDs; better diagnoses of ARDs; and, epidemiological investigation of individual cases of ARDs. In addition to establishing appropriate links between exposures and ARDs; medical research into the treatment of mesothelioma to improve the quality of life and the survival rate, effective early intervention followed by early detection of ARDs is essential.

Identifying barriers that impact the uptake of policies and programmes for the elimination of ARDs, and standardise and harmonise ways these can be addressed is critical [19,20]. Such barriers include business, economic, and political interests; lack of leadership and commitment; gaps in data; and insufficient support and funding for transition from asbestos to safe alternatives. Completion and maintenance of national profiles and development, implementation, and monitoring of a National Program for the Elimination of Asbestos-Related Diseases, including time bound, measurable targets in each country are essential to effectively address barriers and provide facilitators for a strategic approach to addressing ARDs [6].

It should be noted that some contexts in interview statements made may not be precise, as not all of the participants and interviewees were fluent in both English and Russian and should be viewed as a limitation of the study. Assistance of English/Russian interpreters were used to support the data and information gathering for both the interviews and the workshop to allow participants to fully engage and enhance the accuracy of communication.

## 5. Conclusions

The substantial and increasing costs associated with the continued production and use of asbestos far outweigh the short-term and often only local economic benefits, and use of sound and convincing scientific and economic evidence in combination can be used to make convincing arguments to support the uptake of evidence-based policy actions. Key stakeholders, including WHO needs to be empowered to take on a leadership role to advance the uptake of evidence-informed policies and programmes to further the 2030 Agenda for achieving the United Nations Sustainable Development Goals that includes goal number 12, “Responsible Consumption and Production” [21]. This should include the important role of forming large consensus stakeholder groups, nationally and internationally, with a common vision to design, implement, and monitor effective policy solutions. Noting that, banning the production and use of all forms of asbestos, as recommended by the International Labour Organization and WHO, continues to be the most efficient and proven evidence-based strategy to eliminate ARD’s.

## Figures and Tables

**Table 1:** Specific suggestions for partnerships to support elimination of asbestos related diseases (ARDs).

Facilitator Method	Partners *	Specific Action
Leadership	WHO Regional Offices International Labour Organization (ILO)	Call for a complete ban on the use and production of all types of asbestos in the WHO European Region and link to a global ban.
Leadership	European Commission (EC)	Explore means to put more asbestos related legislation and or stringent conditions in place as part of country requirements to join the EU.
Data	WHO European Region Member States Organization for Economic Co-operation and Development (OECD)	Collect and publish data through public report cards with performance indicators related to asbestos, including costs assessments by country.
Management and coordination	WHO European Region Member States	Support countries to develop and maintain regularly, their National Programme for the Elimination of ARDs (NPEAD) with existing tools.
Capacity	WHO European Region Member States WHO Regional Offices Non Governmental Organisations (NGOs) European Environment Agency (EEA) European Agency for Safety and Health at Work (EU-OSHA)	Support capacity building with prevention courses, drafting legislation, and good practice guidelines such as diagnosis criteria of ARDs, safe and economical identification, removal, replacement and waste management of asbestos.
Capacity	WHO European Region Member States WHO Regional Offices European Environment Agency (EEA) European Agency for Safety and Health at Work (EU-OSHA) International Labour Organization (ILO) Organization for Economic Co-operation and Development (OECD) Non Governmental Organisations (NGOs) Asian Development Bank (ADB) World Bank (WB)	Support sharing of country case examples and enhanced data collection and data monitoring, including health evaluation and using methodologies to other health end points that are standardised globally to demonstrate co-benefits to other sectors such as environment, labour, justice, and tourism to address health issues.

Table contains a listing of facilitator methods, partners can undertake through specific actions listed (* The list of partners is restricted due to limited space.).
